# Expression of a Serine Protease Gene *prC* Is Up-Regulated by Oxidative Stress in the Fungus *Clonostachys rosea*: Implications for Fungal Survival

**DOI:** 10.1371/journal.pone.0013386

**Published:** 2010-10-14

**Authors:** Cheng-Gang Zou, Yong-Fang Xu, Wen-Jing Liu, Wei Zhou, Nan Tao, Hui-Hui Tu, Xiao-Wei Huang, Jin-Kui Yang, Ke-Qin Zhang

**Affiliations:** 1 Laboratory for Conservation and Utilization of Bio-Resources & Key Laboratory for Microbial Resources of the Ministry of Education, Yunnan University, Kunming, Yunnan, China; 2 Center for Human Reproduction, The First People's Hospital of Yunnan Province, Kunming, Yunnan, China; Technical University of Denmark, Denmark

## Abstract

**Background:**

Soil fungi face a variety of environmental stresses such as UV light, high temperature, and heavy metals. Adaptation of gene expression through transcriptional regulation is a key mechanism in fungal response to environmental stress. In *Saccharomyces cerevisiae*, the transcription factors Msn2/4 induce stress-mediated gene expression by binding to the stress response element. Previous studies have demonstrated that the expression of extracellular proteases is up-regulated in response to heat shock in fungi. However, the physiological significance of regulation of these extracellular proteases by heat shock remains unclear. The nematophagous fungus *Clonostachys rosea* can secret an extracellular serine protease PrC during the infection of nematodes. Since the promoter of *prC* has three copies of the stress response element, we investigated the effect of environmental stress on the expression of *prC*.

**Methodology/Principal Findings:**

Our results demonstrated that the expression of *prC* was up-regulated by oxidants (H_2_O_2_ or menadione) and heat shock, most likely through the stress response element. After oxidant treatment or heat shock, the germination of conidia in the wild type strain was significantly higher than that in the *prC* mutant strain in the presence of nematode cuticle. Interestingly, the addition of nematode cuticle significantly attenuated the production of reactive oxygen species (ROS) induced by oxidants and heat shock in the wild type strain, but not in *prC* mutant strain. Moreover, low molecule weight (<3 kD) degradation products of nematode cuticle suppressed the inhibitory effect of conidial germination induced by oxidants and heat shock.

**Conclusions/Significance:**

These results indicate that PrC plays a protective role in oxidative stress in *C. rosea*. PrC degrades the nematode cuticle to produce degradation products, which in turn offer a protective effect against oxidative stress by scavenging ROS. Our study reveals a novel strategy for fungi to adapt to environmental stress.

## Introduction

In the soil environment, fungi likely face many potential adverse environmental conditions such as osmotic shock, high temperature, heavy metals, and UV irradiations. To survive and grow in such stressful conditions, fungi have evolved multiple defense systems with some relying on enzymatic actions. One of responses is characterized by a rapid increase in transcriptional expression of stress response element (STRE) gene upon stress [1], [2]. In *Saccharomyces cerevisiae*, the STRE genes are activated by two transcription factors Msn2 and Msn4 in response to stress such as oxidative stress, heat shock, osmotic shock, and glucose starvation. Under normal non-stressful conditions, Msn2 and Msn4 typically reside in the cytoplasm. During stress, these two proteins are translocated into the nucleus, and bind to the promoters of STRE genes. For example, the induction of antioxidant gene catalase is largely dependent on Msn2/Msn4p in response to heat shock and H_2_O_2_ [1].

Numerous fungi secrete extracellular proteases when grown in a medium containing proteins [3]. Interestingly, the expression of secreted extracellular proteases can be induced by heat shock in fungi [4], [5]. For instance, using serial analysis of global gene expression, Steen et al [4] have found that the expression of a subtilisin-like serine protease is up-regulated 3.4-fold by heat shock in the pathogenic fungus *Cryptococcus neoformans*. Likewise, Nevarez et al [5] have reported that the expression of an aspartyl protease is induced in the fungus *Penicillium glabrum* under thermal stress. However, the mechanism underlying heat shock-mediated up-regulation of extracellular proteases remains unknown. Nematophagous fungi have been used as biological control agents against nematodes parasitic on plants and animals. Accumulated evidence indicates that subtilisin-like serine proteases secreted by nematophagous fungi play an essential role during the infection of nematodes [6], [7], [8], [9], [10]. Recently, we isolated and purified a subtilisin-like extracellular protease (designated PrC) from the nematophagous fungus *Clonostachys rosea* (syn. *Gliocladium roseum*) [10]. As a major extracellular serine protease in *C. rosea*, PrC can quickly hydrolyse proteins of the nematode cuticle. To investigate the regulation of *prC* expression, we have cloned the promoter sequence of *prC* (the GenBank accession no. FJ966246)[11]. The promoter region contains putative transcription control sites for nitrogen regulation (5′-GATA), carbon regulation (5′-SYGGRG), pH regulation (5′-GCCARG), and STRE (5′-AGGGG). Our results have demonstrated that the expression of *prC* is regulated by pH [12] and nitrogen sources [11], but not by carbon sources [12]. Given that the promoter of *C. rosea prC* contains three consensus STRE sites, this study investigated the effect of environmental stress on the expression of *prC*. Our results indicated that oxidants and heat shock up-regulated the expression of *prC* at the transcriptional level.

## Results

### *prC* expression is induced by oxidants and heat shock

**Figure 1:**
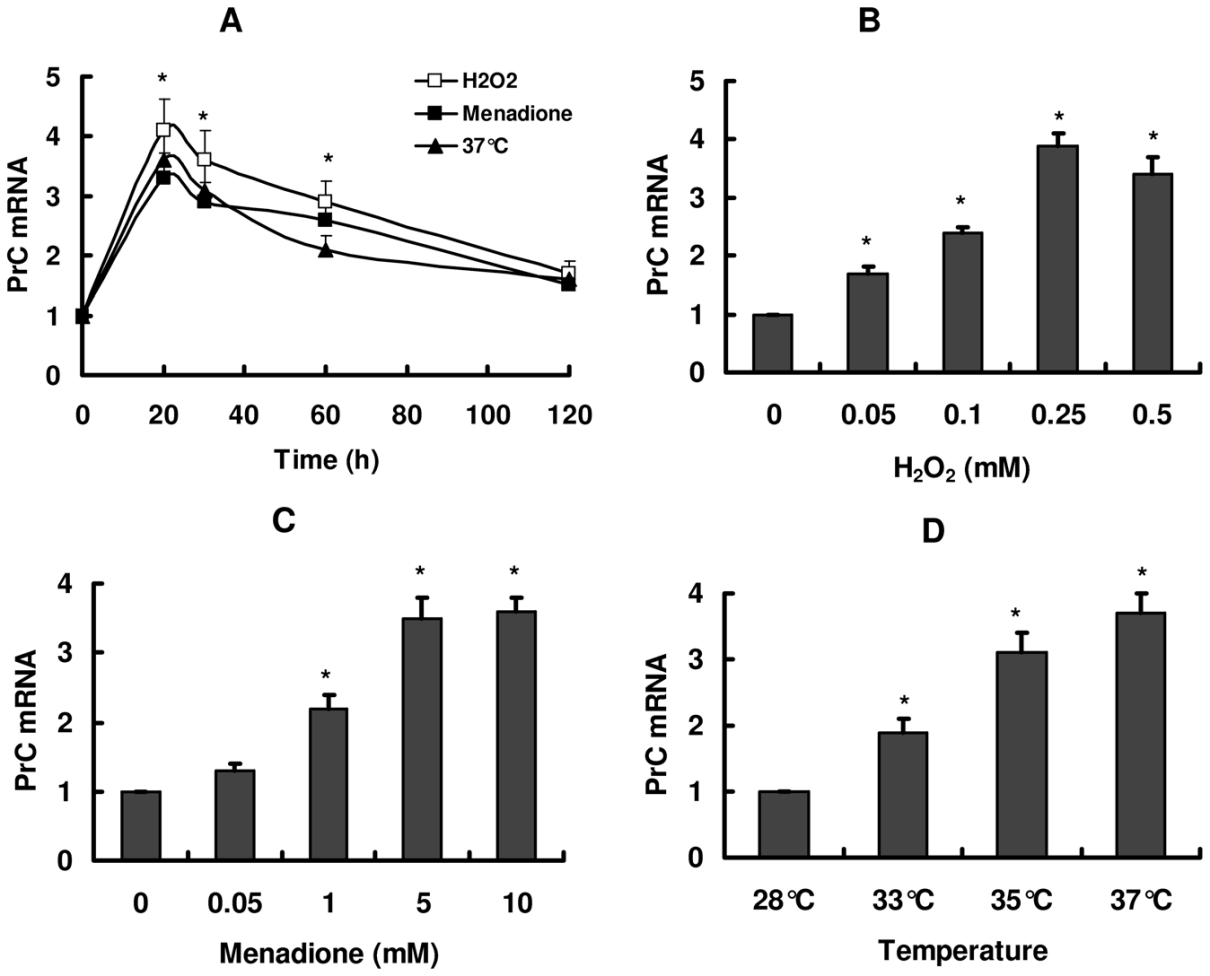
Oxidants and heat shock induce expression of *C. rosea prC*. A. Conidia of *C. rosea* were suspended in YNB medium containing 1% glucose as the carbon source at 28°C. After addition of 0.5 mM of H_2_O_2_ or 5 mM of menadione, or shifting cells to 37°C, cells were removed at the indicated time intervals. B. Conidia of *C. rosea* were incubated with H_2_O_2_ for 20 min. C. Conidia of *C. rosea* were incubated with menadione for 20 min. D. Conidia of *C. rosea* were suspended in YNB medium containing 1% glucose at different temperature for 20 min. The total RNA was extracted at the indicated time periods. The mRNA levels were detected by real-time PCR. All results are standardized to the levels of β-tubulin and are the means ± SD of five independent experiments. ^*^ *P*<0.05 versus control (without oxidants or 37°C).

To investigate the effect of environmental stress on the expression of *prC*, conidia of *C. rosea* were treated by oxidants (H_2_O_2_ or menadione), heat shock, hyperosmotic shock and heavy metal ions (cadmium and lead). As shown in Fig. 1A, *prC* expression increased by oxidant treatments, and reached a maximal level in 20 min. The effect of these oxidants on *prC* expression was found to be dose-dependent. 0.5 mM of H_2_O_2_ and 5 mM of menadione were sufficient to achieve a maximal effect (Fig. 1B and C). The expression of *prC* was also rapidly induced after shifting cells from 28°C to higher temperature (33–37°C) (Fig. 1D) and increased to peak levels by 20 min treatment (Fig. 1A). In contrast, hyperosmotic shock (0.5–1.5 M NaCl) and treatment with cadmium (0.01–0.1 mM) and lead (0.02–0.2 mM) did not affect the expression of *prC*. Similar results were obtained from the mycelia of *C. rosea* (data not shown).

**Figure 2:**
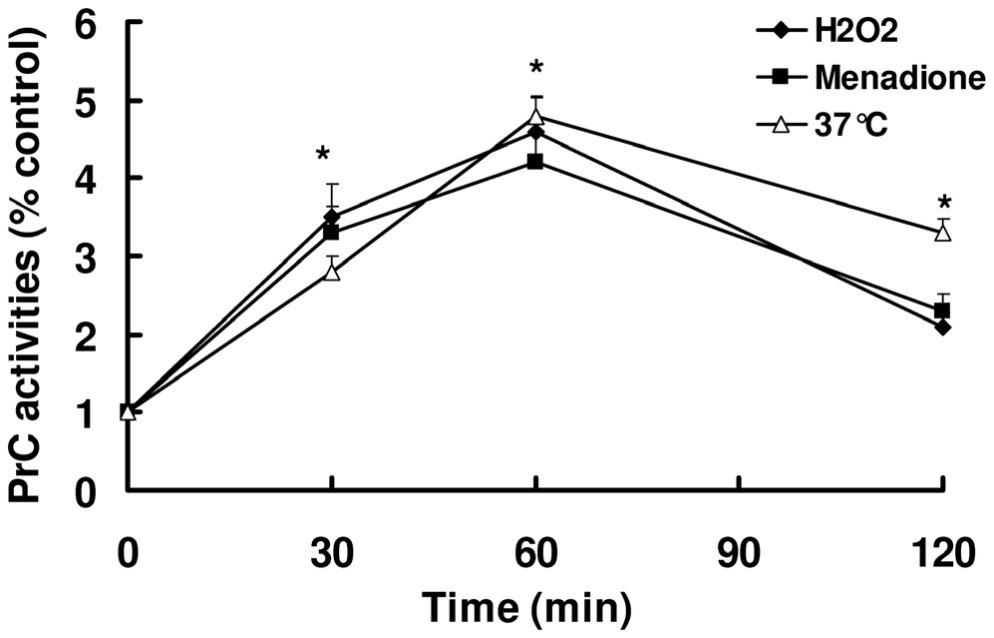
Oxidants and heat shock increase PrC activity in medium. Conidia of *C. rosea* were suspended in YNB medium containing 1% glucose as the carbon source at 28°C. After addition of 0.5 mM of H_2_O_2_ or 5 mM of menadione, or shifting cells to 37°C, the media were collected at the indicated time intervals and the supernatant was filtered. The activities of PrC in filtered supernatant were determined using succinyl-(alanine) 2-proline-phenylalanine-p-nitroanilide as the substrate. * *P*<0.05 versus control.

Since PrC is a secreted protease, we investigated whether the elevation of mRNA levels by oxidants and heat shock would lead to an increase in PrC activities in the culture medium. As shown in Fig. 2, PrC activities in the culture medium (YNB + glucose) were significantly increased after conidia of *C. rosea* were treated by oxidants (H_2_O_2_ or menadione) and heat shock for 30 min. Consistent with the results of mRNA levels, hyperosmotic shock and heavy metal ions (cadmium and lead) did not affect the activities of PrC in the culture medium.

### STRE in *prC* promoter is essential for induction of *prC* expression by stress

**Figure 3:**
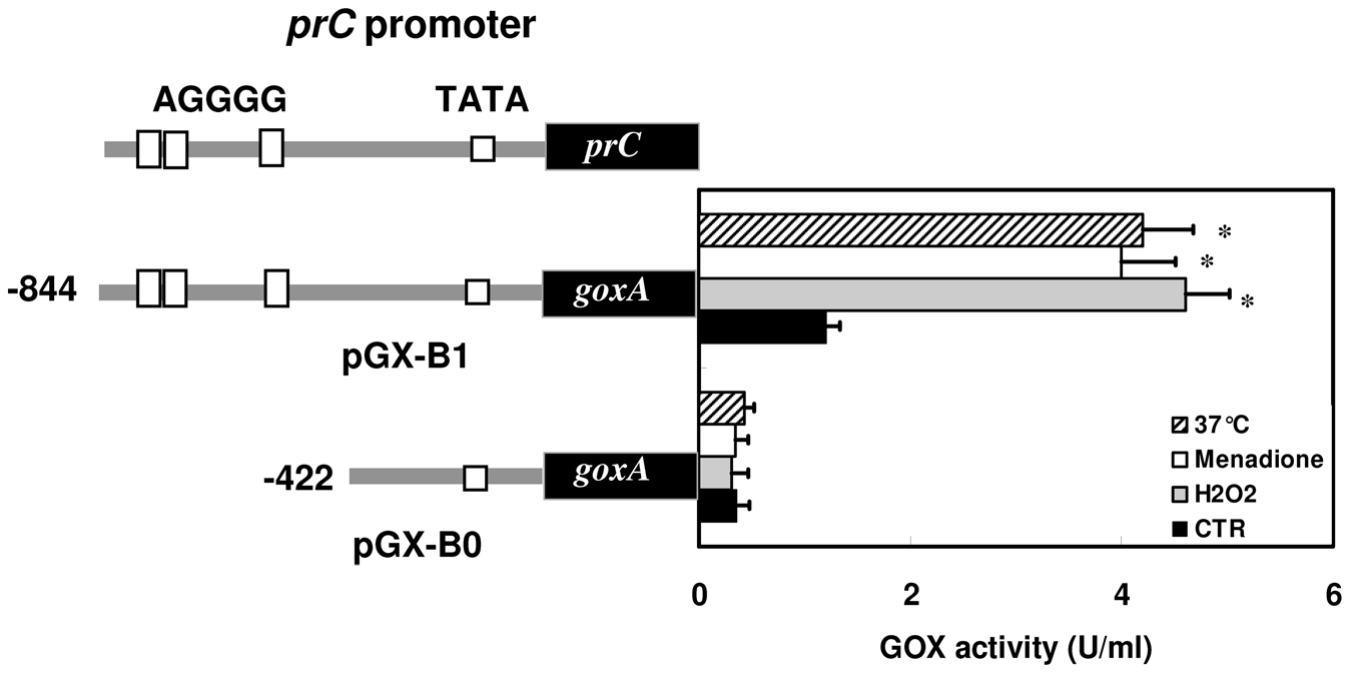
Reporter gene assay. *prC* promoter-*goxA* reporter vectors pGX-B1 and pGX-B0 were respectively transformed into protoplasts. The strains were grown in YNB medium containing 1% (w/v) glucose at 28°C. After the addition of 0.5 mM of H_2_O_2_ or 5 mM of menadione, or shifting cells to 37°C, the media were collected. Glucose oxidase (GOX) enzyme activity (U/ml of culture filtrate) was determined in at least three independent experiments. * *P*<0.05 versus control (CTR).

The up-regulation of *prC* expression by oxidants and heat shock may be the result of transcriptional activation of this gene. We thus constructed the *prC*-promoter glucose oxidase (*goxA*) reporter vectors. Of the two vectors, pGX-B1 contains 844 bp of the 5′ promoter region, which includes the three consensus STRE sites. pGX-B0 contains 422 bp of the 5′ promoter region and has no STRE site. Transformants were grown in YNB medium using glucose as the carbon source. As shown in Fig. 3, a significant increase in glucose oxidase activity was observed after oxidant treatment and heat shock only in the transformants with pGX-B1, not with pGX-B0. These results indicated that the region −844 to −422 containing three STRE sites was involved in the regulation of *prC* expression upon environmental stress.

### *prC* is required for protection against stress-induced inhibition of conidial germination in the presence of nematode cuticle

**Figure 4:**
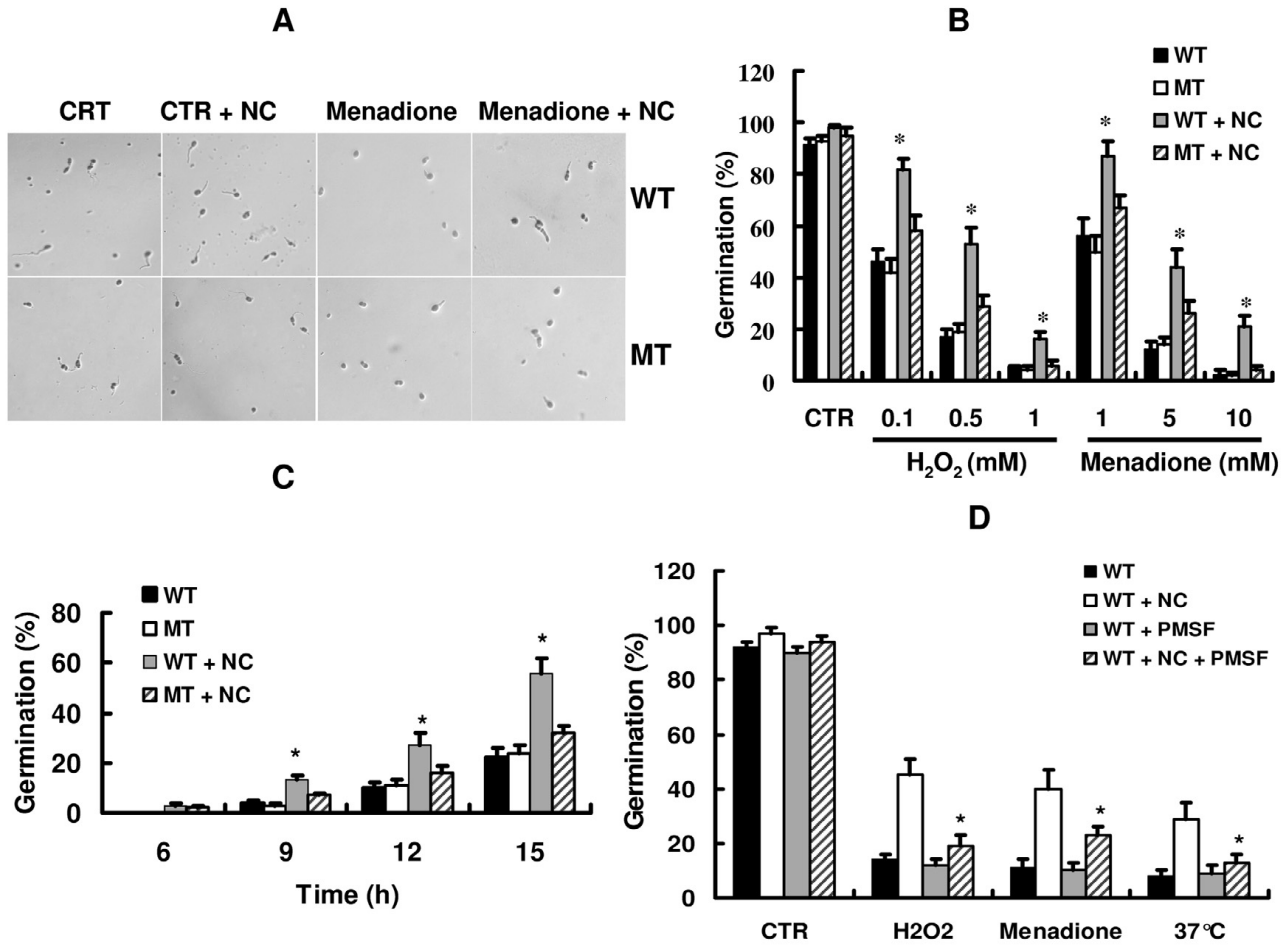
The *prC* mutant is more sensitive to oxidants and heat shock. A. Microscopic examination (×100) of germinated conidia after 9 h at 28°C in YNB agar in the presence or absence of menadione (5 mM) or nematode cuticle (NC) (1 mg/ml). B. Germination frequency of the wild-type (WT) and the *prC* mutant (MT) strains under oxidative stress conditions. Conidia of *C. rosea* were inoculated onto the YNB agar at 28°C in the presence or absence of nematode cuticle (1 mg/ml) or oxidants (menadione or H_2_O_2_). Results are the means ± SD of five independent experiments. *P<0.05 versus wild type. C. Germination frequency of the wild-type and the *prC* mutant strains under heat shock conditions. Conidia were inoculated onto the YNB agar at the different temperatures in the presence or absence of nematode cuticle (1 mg/ml). Results are the means ± SD of five independent experiments. * *P*<0.05 versus WT. D. PMSF inhibited the protective effect of nematode cuticle (1 mg/ml) on conidial germination in the wild-type strain. The concentrations of H_2_O_2_, menadione, and PMSF were 0.5 mM, 5 mM, and 1 mM, respectively. * *P*<0.05 versus WT+NC.

Previously, we found that disruption of *prC* did not influence the germination of conidia and mycelial growth in the presence or absence of nematode cuticle [11]. To investigate whether *prC* mutant might be hypersensitive to oxidants and heat shock, we compared the germination rates of conidia in the wild type and *prC* mutant strains. 90% of conidia in both the wild type and the *prC* mutant strains germinated within 12 h after inoculation onto YNB agar without any stress. However, treatments with oxidants markedly inhibited conidial germination (Fig. 4A). In the absence of nematode cuticle, the germination rates of conidia in the *prC* mutant strain were similar to those of the wild type strain after treatment with H_2_O_2_ (0.1, 0.5, 1 mM) or menadione (1, 5, 10 mM) (Fig. 4B). In contrast, after the addition of nematode cuticle, the conidial germination rates of the wild type strain were significantly higher than those of the *prC* mutant stain in the presence of H_2_O_2_ (0.1, 0.5, 1 mM) or menadione (1, 5, 10 mM). These results suggested that PrC activity in the medium was probably critical for the protection of cells against oxidative stress. To test this hypothesis, an inhibitor of serine protease, phenylmethylsulfonyl fluoride (PMSF) was applied. We found that PMSF (1 mM) alone did not markedly influence the germination of conidia in the wild type strain. However, the pre-treatment of *C. rosea* with PMSF (1 mM) attenuated the protective effect of nematode cuticle against oxidants-induced inhibition of conidial germination in the wild type strain (Fig. 4D).

Neither the wild-type nor the *prC* mutant strains could germinate at 42°C even in the presence of nematode cuticle. Thus, we performed experiment for the germination of conidia at 37°C. The conidial germination was markedly suppressed in both the wild-type and the *prC* mutant strains at 37°C. The germination rates of conidia in the *prC* mutant strain were similar to those in the wild-type strain in the absence of nematode cuticle at 37°C. In contrast, 56% of conidia in the wild-type strain germinated 15 h after the addition of nematode cuticle at 37°C, whereas only 32% of conidia in the *prC* mutant strain germinated under the same conditions (Fig. 4C). Pre-treatment with PMSF significantly inhibited the germination of conidia in the wild type strain in the presence of nematode cuticle at 37°C (Fig. 4D).

### PrC plays a role in scavenging reactive oxygen species by oxidants and heat shock

**Figure 5:**
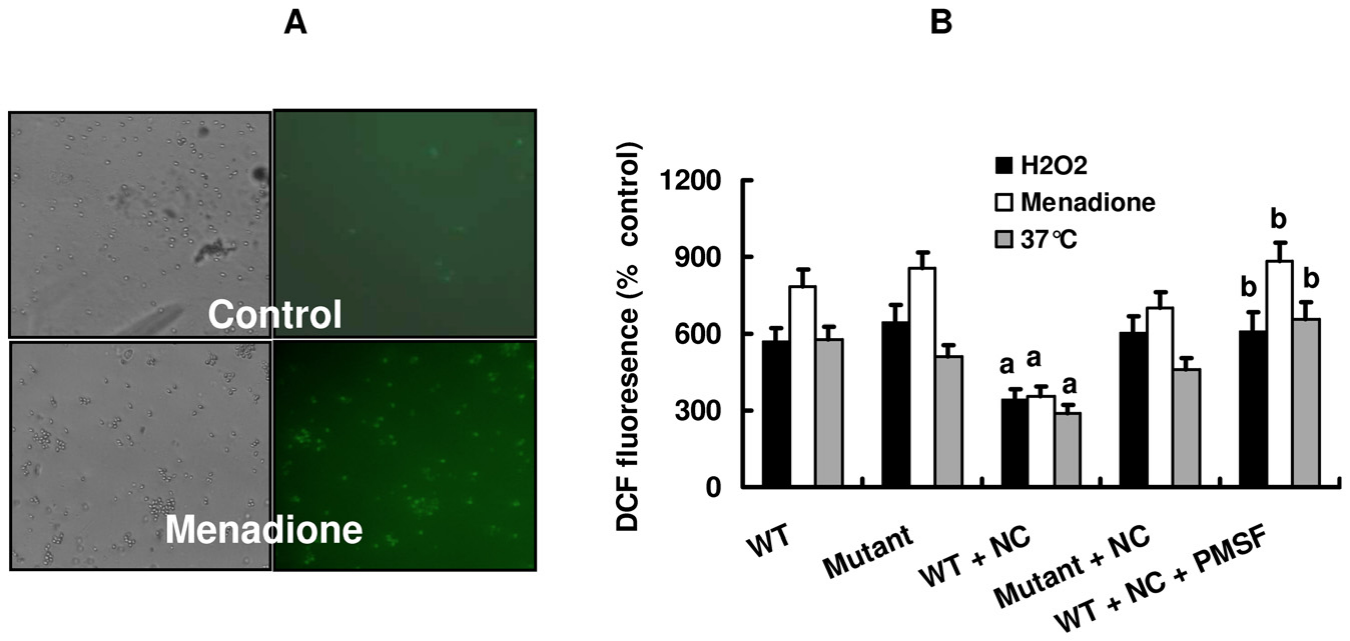
ROS production analysis. A. Approximately 10^6^ conidia were pre-incubated with H_2_DCF-DA (50 µg/ml) for 2 h, then were washed twice with PBS and resuspended in YNB medium. After the conidia were incubated with menadione (5 mM) for 2 h, conidia were plated on YNB agar plates and visualized using a fluorescence microscope. Control: without menadione. B. DCF fluorescence intensity was measured by using a Cytofluor II fluorescent plate reader. The concentrations of H_2_O_2_, menadione and PMSF were 0.5 mM, 5 mM, and 1 mM, respectively. ^a^ *P*<0.05 versus WT; ^b^ *P*<0.05 versus WT+NC.

To study whether the protective effect of PrC was due to its antioxidant property, we determined the levels of reactive oxygen species (ROS) in the conidia of *C. rosea* by using a fluorescence dye 2′,7′-dichlorodihydrofluorescein diacetate (H_2_DCFDA). H_2_DCFDA can easily diffuse into cells and is trapped after cleavage of H_2_DCFDA to H_2_DCF by intracellular esterases. Oxidation of H_2_DCF by intracellular ROS leads to the formation of a fluorescent product, DCF [13]. For this essay, *C. rosea* conidia were collected and stained with H_2_DCFDA (50 µg/ml) for 2 h. Enhanced ROS production in the conidia stimulated by H_2_O_2_ (0.5 mM), menadione (5 mM) or heat shock (37°C) was observed in DCF fluorescence both in the wild type and *prC* mutant strains (Fig. 5A & B). The ROS production induced by H_2_O_2_, menadione or heat shock was markedly reduced by the addition of nematode cuticle in the wild type strain, but not in the *prC* mutant strain (Fig. 5B). Pre-treatment with PMSF significantly suppressed the inhibitory effect of nematode cuticle on the formation of ROS induced by H_2_O_2_, menadione or heat shock (37°C) (Fig. 5B).

### The degradation products of nematode cuticle antagonize the inhibitory action on conidial germination

**Figure 6:**
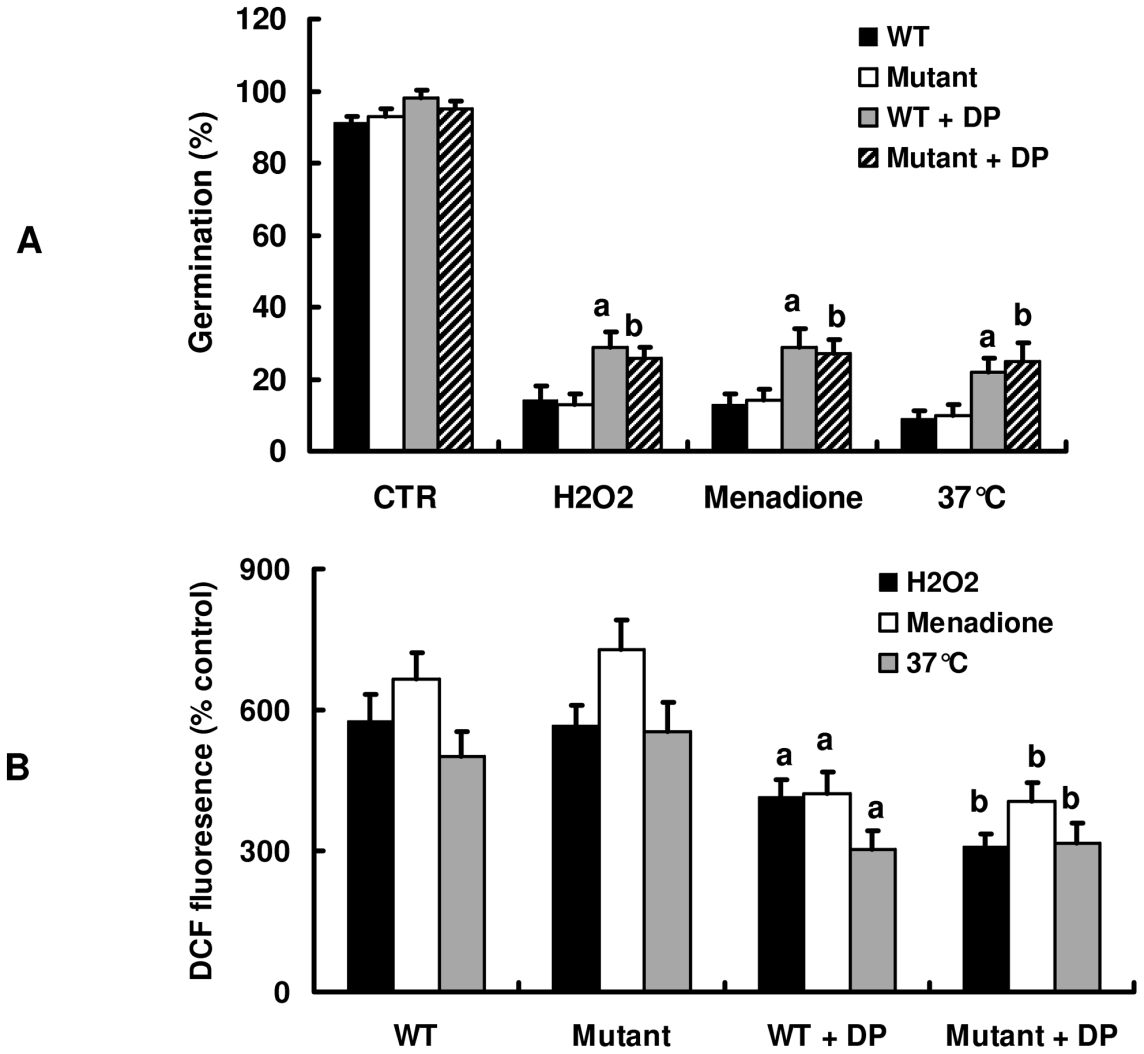
The degradation products of nematode cuticle induce *prC* expression. A. Germination frequency of the wild-type (WT) and the *prC* mutant strains for 9 h in glucose-containing YNB agar in the presence or absence of the degradation products (DP) (50 µg/ml) or 0.5 mM of H_2_O_2_ or 5 mM of menadione at 28°C. In some cases, conidial germination was determined without oxidants at 37°C. Results are the means ± SD of five independent experiments. ^a^ *P*<0.05 versus WT; ^b^ *P*<0.05 versus mutant. B. Conidia of the wild-type and the *prC* mutant strains in YNB medium were incubated with 0.5 mM of H_2_O_2_ or 5 mM of menadione in the presence or absence of the degradation products (DP) (50 µg/ml) at 28°C. In some case, conidia were incubated at 37°C. DCF fluorescence intensity was measured by using a Cytofluor II fluorescent plate reader. ^a^ *P*<0.05 versus WT;^b^ *P*<0.05 versus mutant.

The expression of extracellular serine proteases is strongly induced by nematode and insect cuticles in nematophagous and entomopathogenic fungi, respectively [8], [9], [14], [15]. Recently, we demonstrated that the degradation products of nematode cuticle with molecule weights less than 3 kD significantly induced the expression of *prC* in *C. rosea* [11]. Unlike oxidants and heat shock, the degradation products induced *prC* expression 12 h after incubation. We thus investigated the direct effect of the degradation products with molecule weights less than 3 kD on the conidial germination inhibition. As shown in Fig. 6A, the addition of the degradation products (50 µg/ml) markedly suppressed the inhibitory effect of oxidants and heat shock on conidial germination in both the wild type and the *prC* mutant strains. Furthermore, the degradation products also reduced the production of ROS induced by H_2_O_2_ (0.5 mM), menadione (5 mM) or heat shock (37°C) (Fig. 6B). Unlike nematode cuticle, the effect of its degradation products on conidial germination and ROS scavenging were not affected by pre-treatment of PMSF with *C. rosea* (data not shown).

2,2-Diphenyl-1-picrylhydrazyl (DPPH) and 2,2′-Azinobis(3-ethylbenzothiazoline- 6-sulfonic Acid) (ABTS^+^) radicals have been commonly used to evaluate antioxidant capacity [16]. To study whether the protective effect of the nematode cuticle and its degradation products was due to its antioxidant property, the scavenging activity of free radicals of the degradation products was determined. However, neither the nematode cuticle nor its degradation products showed any DPPH and ABTS^+^ scavenging ability (data not shown).

## Discussion

In this study, we demonstrated that the subtilisin-like extracellular protease *prC* was regulated by environmental stress, such as oxidants and heat shock. Increased expression of secreted extracellular proteases by heat shock has been previously reported [4], [5]. However, the physiological significance of regulation of these extracellular proteases by heat shock remains largely unknown. The STRE site is a universally conserved stress element across fungi [17], [18], [19], [20]. In *S. cerevisiae*, the STRE genes require the general stress factors Msn2 and Msn4. However, fungal Msn2p and Msn4p, which are classical zinc finger proteins, are predominantly found in the ascomycete family [21]. It is still not clear whether functional Msn2/4 orthologues exist in other ascomycetous fungi apart from *S. cerevisiae*. In *Candida glabrata*, the orthologues CgMsn2 and CgMsn4 function as stress-responsive transcription factors, which mediate the expression of numerous genes involved in defense against oxidative and osmotic stress as well as heat shock [22]. The *Trichoderma atroviride SEB1* gene encoding an STRE-binding protein is also involved in the response to high osmolarity stress [23]. In *S. cerevisiae*, the expression of approximately 180 genes responding to heat shock or H_2_O_2_ treatment has been found controlled by Msn2 and Msn4 [1]. A remarkable feature of the expression of these genes in response to heat shock and oxidants is that the induction in transcript abundance is very rapid and largely transient. Most of these genes are up-regulated within 15–20 min. In the present study, we found that the induction of *prC* expression by oxidants or heat shock occurred rapidly and reached a maximal level in 20 min. The promoter activity assays demonstrated that the *prC* promoter could be activated by heat shock or oxidants treatment, indicating that the regulation exerted by oxidative stress on *prC* expression appeared to be transcriptional. The region −844 to −422 containing three STRE elements was critical in the activation of *prC* promoter by oxidants or heat shock, suggesting that *prC* expression is likely mediated through the STRE elements.

The deletion of *prC* did not influence the germination of conidia and mycelial growth, suggesting that this gene is dispensable in fungal growth under normal conditions [11]. In contrast, the deletion of *prC* or the inhibition of PrC activity by PMSF rendered conidia more sensitive to oxidants or heat shock in the presence of nematode cuticle, but not in the absence of cuticle. These results implicate that the existence of nematode cuticle is important for PrC to protect cells against environmental stress in the wild type strain. The fact that the presence of nematode cuticle could protect oxidants or heat shock against germination inhibition suggests that the degradation products of nematode cuticle are probably involved in the regulatory process. Our recent study have demonstrated that the degradation products of nematode cuticle with molecule weights less than 3 kD are capable of inducing *prC* expression in *C. rosea* [11]. Unlike oxidative stress, the degradation products induce *prC* expression 12 h after incubation. In the present study, we found that the degradation products of nematode cuticle with molecule weights less than 3 kD were effective in attenuating the inhibitory effect of oxidants or heat shock on the conidial germination. These data indicate that PrC functions as an indirect participant in protecting cells from oxidants or heat shock. It is worthy of note that the conidial germination of the *prC* mutant strain was similar to that of the wild-type strain in the presence of nematode cuticle under normal conditions (Fig. 4). Thus, cuticle degradation products are not likely to play a critical role in normal conidial germination.

How can the degradation products protect cells from oxidants and heat shock? It is well known that heat shock significantly induces oxidative stress in both yeast and filamentous fungi [24], [25]. The production of ROS induced by oxidants or heat shock was markedly inhibited by the degradation products from nematode cuticle, suggesting that the degradation products are probably involved in scavenging ROS in *C. rosea*. However, our *in vitro* results indicated that the degradation products itself had no antioxidant properties. Thus, the degradation products probably entered into *C. rosea* cells and initiated a signal transduction pathway to detoxify ROS. In *C. albicans*, extracellular aspartic proteinases are secreted to degrade host tissues, leading to the production of peptides from the degradation of proteins [26]. These peptides can be taken up into the cell by oligopeptide transporters and activate a signal transduction event to further induce the expression of aspartic proteinases [27]. However, how these peptides induce aspartic proteinases remains unknown.

In summary, we have demonstrated that the expression of *prC* is rapidly induced after the fungus *C. rosea* is exposed to oxidants and heat shock. The induction is likely mediated through the STRE elements, leading to more PrC secreted into medium. PrC degrades the nematode cuticle, resulting in the release of degradation products, which in turn offer a protective effect against oxidative stress by scavenging ROS. This work identifies a new function for PrC in *C. rosea*, i.e. participation in the detoxification of ROS.

## Materials and Methods

### Fungal strains and stress treatment conditions

The wild-type strain 611 of *C. rosea* used in this study was previously described by Li et al. [10]. Fungal cultures were maintained on yeast nitrogen base (YNB) medium containing 0.67% (w/v) YNB (Difco Laboratories, Sparks, MD) and 1% (w/v) glucose in the presence or absence of nematode cuticle. Media were buffered with 50 mM Tris-HCl at pH 8.

For mRNA and enzyme assays with conidia, 10^7^ conidia/ml were suspended in the YNB medium. One of the indicated compounds was added to the cells with shaking at 150 rpm at 28°C, respectively. In some experiments, the conidia suspensions were given heat shock at 33–37°C. 1 ml of the cell suspensions were removed at different times. Cells were then harvested by centrifugation for mRNA detection. The supernatant was collected for enzyme assay. For mRNA and enzyme assays with mycelia, the fungus was grown in the YNB medium with shaking at 150 rpm for five days at 28°C with an initial concentration of 10^6^ conidia/ml. Following heat shock, or oxidative shock, or hyperosmotic shock, an aliquot of each culture was withdrawn for mRNA detection or enzyme assay.

### Enzyme assay

Nematode cuticle was extracted according to the method described by Cox et al. [28]. *C. rosea* conidia or mycelia were grown in the YNB medium as described above. After treatment at the indicated times, the media were collected and centrifuged at 6000 × g for 5 min. The supernatant was filtered through Waterman paper. The subtilisin-like protease activities of PrC in filtered supernatant were determined using succinyl-(alanine) 2-proline-phenylalanine-p-nitroanilide as the substrate. The enzyme activities are given in the increase of A405/ml/min at 37°C.

### Quantitative real-time RT-PCR analysis

Total RNA from *C. rosea* conidia or mycelia was isolated using the Trizol reagent (Invitrogen, Carlsbad, CA). Random-primed cDNAs were generated by reverse transcription of the total RNA samples with SuperScript II (Invitrogen). A real time-PCR analysis was performed with the ABI Prism 7000 Sequence Detection System (Applied Biosystems, Foster City, CA) using SYBR® Premix-Ex TagTM (Takara, Dalian, China). β-Tubulin was used for an internal control. The primers used for PCR were as follows: *PrC*: 5′-GCC AAC GCT GCC AAC TAC TC-3′ (F), 5′-TGT AGG CGG AGA GGA CGG A-3′ (R); β-tubulin: 5′- CAT CTT CAG ACC GGT CAG TG-3′ (F), 5′- AAG TAG ACG TTC ATG CGC TC -3′ (R).

### Reporter gene construction and fungal transformation

The plasmid pGX was created for reporter gene analysis as described previously [12]. Briefly, an 844 bp fragment of the *prC* promoter region containing 5′-AGGGG-3′ STRE sites was amplified by PCR with genomic DNA as template and using primers PrCF1 (5′- CGG TAC CGG TCT CTT TTC TAG TTT G-3′), PrCR1 (5′- GGG GAT CCT TTG TTG CTT GGT G -3′) that contained KpnI and BamHI restriction sites. The fragment was subcloned into plasmid pGX, resulting in pGX-B1. An 422 bp fragment of the *prC* promoter region without 5′-AGGGG-3′ was amplified by PCR with *prC* genomic DNA as template and using primers PrCF2 (5′- GAA GAT CTC TGT GGG TTC GTC GTA -3′), PrCR2 (5′- GCT CTA GAC GGT TTG TTG CTT GGT GAA G -3′) that contained BglII and XbaI restriction sites. The fragment was subcloned into plasmid pGX, resulting in pGX-B0.

The plasmids pGX-B1 and pGX-B0 were introduced into protoplasts of *C. rosea* by co-transformation with plasmid pANGH3 containing a green fluorescence protein gene following the protoplast-based protocol, respectively [29]. Transformants expressing GFP were observed under a fluorescence microscope. The positive clones confirmed by PCR were used for reporter gene analysis.

### Glucose oxidase assay

The activity of glucose oxidase was determined as described previously [17]. 0.2 ml of culture supernatant was mixed with 1.8 ml of the assay solution (containing 1.4 mM ABTS (2,29-azino-di-[3-ethylbenzthiazoline sulfonate]) (Sigma, St. Louis, MO), 1.5 U/ml horseradish peroxidase (Sigma), and 250 mM glucose in 100 mM sodium phosphate buffer (pH 5.8). The absorption at 420 nm was measured continuously in a Beckman spectrophotometer. One unit of enzyme activity was defined as the amount of enzyme required to oxidize 1 µmol of glucose per min at 25°C.

### Germination of conidia

For germination assays with menadione, 10^5^ of conidia were inoculated onto 1.5% (w/v) agar plates of YNB containing menadione (0–10 mM) in the presence or absence of nematode cuticle (1 mg/ml). For germination assays with H_2_O_2_, 10^5^ conidia were inoculated onto 1.5% (w/v) agar plates of YNB in the presence or absence of nematode cuticle (1 mg/ml). And then the plates were overlaid with a solution of H_2_O_2_ at concentrations ranging from 0 to 1 mM. For germination assays with heat shock, 10^5^ of conidia were inoculated onto on 1.5% (w/v) agar plates of YNB at the different temperatures in the presence or absence of nematode cuticle (1 mg/ml). After incubation at different times, the rate of germination was determined by examination of at least 25 randomly chosen microscopic fields.

### Isolation of the degradation products of nematode cuticle

Purification of the PrC protein from culture medium followed protocols described previously [10]. After the purified PrC was incubated with the nematode cuticle (1 mg/ml) for 20 min, the reaction was terminated by heat at 100°C for 5 min. The reaction mixture was collected and applied to a centrifugal iCONTM concentrator (MWCO 3 kD, Pierce, Rockford, IL). After centrifuging at 7,500× g for 2 h, the flow-through in the concentrator was collected for further assay.

### Detection of ROS

For detection of ROS, *C. rosea* conidia were collected from PDA agar plates with mild scraping using sterile deionized water and washed twice by centrifugation. The pellet was resuspended in YNB medium to a final concentration of 10^6^ conidia per ml. The conidia were incubated with 2′,7′-dichlorodihydrofluorescein diacetate (50 µg/ml) (Molecular Probes, Eugene, OR) at 28°C. After incubation for 2 hour, the conidia were washed twice with PBS to remove excess probe and resuspended in YNB medium. After treatment with H_2_O_2_ (0.5 mM), menadione (5 mM) or heat shock (37°C) for 3 h in the presence or absence of nematode cuticle and its degradation products, respectively, conidia were plated on YNB agar plates and visualized using a Nikon TE2000-U fluorescence microscope (Nikon Inc., Japan) using 495-nm excitation and 500- to 550-nm emission wavelengths. DCF fluorescence intensity was measured by using a Cytofluor II fluorescent plate reader (excitation 485-nm/emission 530-nm; Millipore Corp., Bedford, MA).

### The measurement of free radical scavenging activity

To determine the antioxidant effect of nematode cuticle and its degradation products *in vitro*, a stable DPPH radical (Sigma) was used. The free radical scavenging activities of nematode cuticle and its degradation products were determined following a previously reported method [16]. In addition, the free radical scavenging activities of nematode cuticle and its degradation products were also judged by measuring reduction of radical ABTS^+^ according to the manufacturer's instructions with the kit GMS10114.4 (Genmed Scientifics Inc., Shanghai, China).
